# A resting EEG study of neocortical hyperexcitability and altered functional connectivity in fragile X syndrome

**DOI:** 10.1186/s11689-017-9191-z

**Published:** 2017-03-14

**Authors:** Jun Wang, Lauren E. Ethridge, Matthew W. Mosconi, Stormi P. White, Devin K. Binder, Ernest V. Pedapati, Craig A. Erickson, Matthew J. Byerly, John A. Sweeney

**Affiliations:** 1 Department of Psychology, Zhejiang Normal University, 688 Yingbin Road, Jinhua, Zhejiang China 321004; 2 Department of Pediatrics, Section of Developmental and Behavioral Pediatrics, University of Oklahoma Health Sciences Center, Oklahoma City, OK USA; 3 Department of Psychology, University of Oklahoma, Norman, OK USA; 4 Clinical Child Psychology Program and Schiefelbusch Institute for Life Span Studies, University of Kansas, Lawrence, KS USA; 5 Department of Psychiatry, Center for Autism and Developmental Disabilities, University of Texas Southwestern Medical Center, Dallas, TX USA; 6 Center for Glial-Neuronal Interactions, Neuroscience Graduate Program, Division of Biomedical Sciences, School of Medicine, University of California, Riverside, CA USA; 7 Department of Psychiatry and Behavioral Neuroscience and Division of Psychiatry, Cincinnati Children’s Hospital Medical Center, Cincinnati, OH USA; 8 Center for Mental Health Research and Recovery, Montana State University, Bozeman, MT USA; 9 Department of Psychiatry and Behavioral Neuroscience, University of Cincinnati, Cincinnati, OH USA

**Keywords:** Fragile X syndrome, EEG, Hyperexcitability, Gamma, Cross-frequency coupling, Top-down modulation

## Abstract

**Background:**

Cortical hyperexcitability due to abnormal fast-spiking inhibitory interneuron function has been documented in *fmr1* KO mice, a mouse model of the fragile X syndrome which is the most common single gene cause of autism and intellectual disability.

**Methods:**

We collected resting state dense-array electroencephalography data from 21 fragile X syndrome (FXS) patients and 21 age-matched healthy participants.

**Results:**

FXS patients exhibited greater gamma frequency band power, which was correlated with social and sensory processing difficulties. Second, FXS patients showed increased spatial spreading of phase-synchronized high frequency neural activity in the gamma band. Third, we observed increased negative theta-to-gamma but decreased alpha-to-gamma band amplitude coupling, and the level of increased theta power was inversely related to the level of resting gamma power in FXS.

**Conclusions:**

Increased theta band power and coupling from frontal sources may represent a mechanism providing compensatory inhibition of high-frequency gamma band activity, potentially contributing to the widely varying level of neurophysiological and behavioral abnormalities and treatment response seen in full-mutation FXS patients. These findings extend preclinical observations and provide new mechanistic insights into brain alterations and their variability across FXS patients. Electrophysiological measures may provide useful translational biomarkers for advancing drug development and individualizing treatments for neurodevelopmental disorders with associated neuronal hyperexcitability.

**Electronic supplementary material:**

The online version of this article (doi:10.1186/s11689-017-9191-z) contains supplementary material, which is available to authorized users.

## Background

Fragile X syndrome (FXS) is a neurodevelopmental disorder resulting from silencing of the fragile X mental retardation gene (*FMR1*) on the X chromosome, leading to reduced production of Fragile X Mental Retardation Protein (*FMRP*) [1] that causes atypical brain development and function. Studies in *fmr1* knockout (KO) mice have shown enhanced activity of metabotropic glutamate receptors [2] and reduced GABAergic transmission [3]. These alterations are believed to cause an imbalance favoring excitation over inhibition in brain neurophysiology [3–5].

Neurophysiological studies can clarify the functional brain consequences of neurochemical and neuroanatomic changes in FXS. *fmr1* KO mice have abnormally high synchrony of neocortical network activity and a threefold higher neuronal firing rate during Up states [6, 7]. *fmr1* KO mice have also shown increased EEG responses to auditory stimuli via in vivo recordings [8–10]. Similarly, enhanced auditory event-related potential (ERP) responses (e.g. N1, P2) and reduced response habituation have been reported in FXS patients [11–14].

Given the model of a neurophysiological imbalance leading to heightened neural excitability and the increased prevalence of seizures in FXS patients and *fmr1* KO mice, and with due consideration of the challenges integrating knowledge from intracranial recordings and clinical data, it is noteworthy that few EEG studies of resting brain function have yet been conducted with FXS patients. To our knowledge there has only been one quantitative study of resting state EEG in FXS presented in two reports [15, 16]. Excessive resting state theta and reduced alpha power in FXS were reported, as well as decreased connectivity in alpha and beta bands but increased connectivity in the theta band. While informative, there were certain limitations to that study. First, the sample size was small (8 FXS patients). Second, the study investigated activity under 50 Hz, which excludes a significant component of the gamma frequency band (30–80 Hz) [17–19]. This limitation is important because gamma band power reflects the level of high frequency spontaneous neural activity and is of special interest for FXS in light of *fmr1* KO mouse studies that have identified abnormalities in fast-spiking inhibitory GABAergic interneurons [7, 20] which are critical generators of gamma power in cell populations [21]. Third, functional connectivity analysis was done using only 28 electrodes; a dense electrode montage can better capture the full pattern of functional connectivity across the neocortex. Fourth, the study was insufficiently powered to identify correlations between resting state oscillatory abnormalities in FXS and measures of clinical symptom severity.

Alpha rhythms are the most dominant oscillation during the resting state and play an inhibitory role in information processing systems [22]. Theta rhythms also reflect top-down inhibitory and organizational influences especially during higher cognitive activity [23], and altered theta-gamma coupling has been linked with cognitive dysfunction in *fmr1* KO mice [24]. As increased gamma activity is believed to be linked to increased neural excitability, examining the relationship of alpha and theta band activity with gamma band activity might provide mechanistic system-level understanding about the altered balance between excitatory and inhibitory activity.

The aim of the present study was to investigate resting state EEG activity in FXS patients focusing on resting EEG power—specifically gamma band power, functional connectivity, and gamma coupling. We hypothesized that resting state EEG power in FXS would be enhanced in both low- and high-frequency bands (theta and gamma) but reduced in middle range frequencies (alpha) relative to healthy controls. Second, we predicted that functional connectivity in FXS would be reduced in long-range connections but increased in short-range connections dominated by gamma band oscillations relative to controls. Third, we predicted that FXS patients would show reduced alpha-to-gamma coupling consistent with reduced top-down alpha-related inhibition on local cortical excitability in sensory systems. Fourth, we hypothesized that altered resting EEG power spectra in FXS individuals would be correlated with social and sensory processing difficulties.

## Methods

### Participants

Twenty-one FXS participants with a full mutation (greater than 200 CGG repeats) (six females, 15 males, mean age = 25.6 years, SD = 11.1, range 12–57) and 21 healthy age-matched controls (six females, 15 males, mean age = 26.4 years, SD = 10.5, range 10–55) participated in this study (Table 1). None had a history of nonfebrile seizures or treatment with anticonvulsant medication. Healthy controls had no known prior diagnosis or treatment for a psychiatric or neurological illness or history of developmental delay in educational achievement. FXS participants taking psychiatric medications were receiving a stable dose for at least 4 weeks prior to participation. Nine FXS participants were receiving one or more psychiatric medications: 5 on antipsychotics, 5 on antidepressants, and 2 on psychostimulants. Treated patients did not differ on reported EEG measures (see Additional file 1: Table S1). Primary analyses were done with the whole sample, with confirmatory analyses done with the male participants.

**Table 1:** Demographic, intellectual, and clinical characteristics of study participants

	FXS *n* = 21				Healthy controls *n* = 21			
	Mean	Std dev	Range		Mean	Std dev	Range	*t* statistic (*df*)
Age	25.6	11.1	12–57	Age	26.4	10.5	10–55	0.24 (40) *p* = 0.809
Full scale IQ	55.1	14.8	47–94	Full scale IQ	106.3	10.7	82–123	12.8 (40) *p* < 0.001
Verbal	2.9	3.2	1–11	Verbal	107.7	11.7	82–124	
Nonverbal	2.0	1.9	1–7	Performance	103.2	11.8	82–125	
SCQ scores	17.7	8.6	2–31					
Sensory Profile	31.6	4.7	24–40					

IQ assessed by Stanford Binet in FXS and estimated using the Wechsler Adult Scale of Intelligence in healthy controls

*SCQ* Social and Communication Questionnaire

The Adolescent and Adult Sensory Profile [25] and the Social Communication Questionnaire (SCQ) [26] were completed for FXS participants by their primary caregiver or close family member. IQ of FXS participants was assessed using the Stanford-Binet Intelligence Scale 5^th^ Ed. [27] which characterizes intellectual ability across a broad ability and age range. IQ of healthy controls was estimated using the briefer Wechsler Abbreviated Scale of Intelligence (WASI) [28] (Table 1). The project was approved by the University of Texas Southwestern Institutional Review Board, and informed consent was obtained from all participants, except when appropriate from a parent with participant assent.

### EEG recordings and preprocessing

Five minutes of continuous EEG data was collected. Participants were comfortably seated while watching a silent video (cartoon movie, standardized across participants), done to facilitate cooperation in the FXS patients as in previous studies [29]. EEG data were recorded with a 128-electrode Biosemi Active Two system at a sampling rate of 512 Hz. Two additional electrodes, positioned near to the electrode POz of the international 10-20 system [30], served as recording reference [common mode sense (CMS) active electrode] and ground. The initial 10 s of recordings were excluded from processing to minimize movement artifacts. Raw EEG data were filtered and transformed to an average reference using the EEGLAB toolbox [31]. High- and low-pass cutoff frequencies were set at 0.5 and 100 Hz; a 60-Hz notch filter was used for removal of power-line noise. Then, EEG data were subjected to Fully Automated Statistical Thresholding for segmentation with 2 s each and EEG Artifact Rejection (FASTER) [32], and artifacts including eye blink, muscle activity, and cardiac activity were removed. This process included 5 steps: (1) outlier channels were identified and replaced with interpolated values in continuous data; (2) continuous data was segmented into 2-s epochs; (3) outlier epochs were removed from participants’ epoch set; (4) spatial independent components analysis was applied to remaining epochs, outlier components were identified (including components that correlated with EOG activity), and data were backprojected without these components; and (5) within an epoch, outlier channels were interpolated.

### EEG power

There was no significant group difference in the number of artifact-free epochs (Epochs_control_ = 138, SD = 5.8; Epochs_FXS_ = 139, SD = 4.7, *p* > 0.05) or number of interpolated channels (Interplolated_channel_control_ = 2.33, SD = 1.2; Interplolated_channel_FXS_ = 2.81, SD = 2.06, *p* > 0.05). For each channel, every 2-s epoch was detrended, tapered with a Hanning window, and transformed in Matlab using a Fourier (FFT) algorithm, yielding Fourier coefficients in 0.5 Hz frequency steps. The Fourier coefficients were then squared to yield power values (uV^2^). To be comparable with previous resting-state EEG reports on FXS [16], we divided our frequencies into six frequency bands of interest as follows: delta (1–3 Hz), theta (4–7 Hz), lower alpha (8–10 Hz), upper alpha (10–12 Hz), beta (13–30 Hz), and gamma (30–80 Hz). Alpha band activity was separated into higher and lower bands because previous studies showed that higher alpha is state dependent and sensitive to arousal while lower alpha is not [33], as well as to permit direct comparison with the previous resting EEG study of FXS [15, 16]. To minimize effects of inter-individual variability in total power, relative power was obtained by computing the fraction of power in each frequency band divided by the sum of power measurements across 1–80 Hz for each frequency band in each of the 128 channels. Group comparisons in relative power (power at specific frequency/total power) between healthy and FXS individuals were performed at every channel. To correct for multiple comparisons and identify significant clusters among channels with group differences in a frequency band, a cluster-based permutation test in the Mass Univariate ERP Toolbox was used for statistical comparisons (5000 permutations [34]).

### Connectivity analysis

Functional connectivity among all electrode pairs was examined separately in each of the six frequency bands of interest using the debiased weighted phase lag index (dbWPLI) [35]. This method minimizes artifacts resulting from spurious inflation of scalp EEG connectivity caused by volume conduction. The dbWPLI calculates an unbiased index of phase synchronization between two time series, weighted by the magnitude of the imaginary component of the cross-spectrum. Compared to a direct phase lag index (PLI), the dbWPLI has minimum sample-size bias and improved ability to detect phase synchronization patterns. The dbWPLI value ranges from 0 to 1, with zero indicating the absence of phase-lagged coupling and one indicating the strongest possible coupling.

EEG data were imported to FieldTrip (Donders Institute for Brain, Cognition and Behaviour, Radboud University, Nijmegen, The Netherlands: http://www.ru.nl/neuroimaging/fieldtrip/) for calculating dbWPLI among all electrode pairs (8128 pairs) at each of the six frequency bands of interest. Then, at each frequency band, the obtained dbWPLI difference between FXS and control groups at each electrode pair was tested using a permutation approach [36]. At each electrode pair, dbWPLI from the 21 FXS and 21 control participants were shuffled and randomly separated into two groups in order to calculate the difference in group-mean dbWPLI for each permutation. This was repeated 5000 times, providing a distribution of dbWPLI values for each electrode pair for comparison with actual group differences, which were considered statistically significant if they exceeded the 95% confidence interval of the distribution of dbWPLI differences at the corresponding electrode pair (two-tailed test). A false discovery rate (FDR) approach was implemented in EEGLAB [31] to control the Type I error rate given the multiple comparisons.

**Fig. 1:**
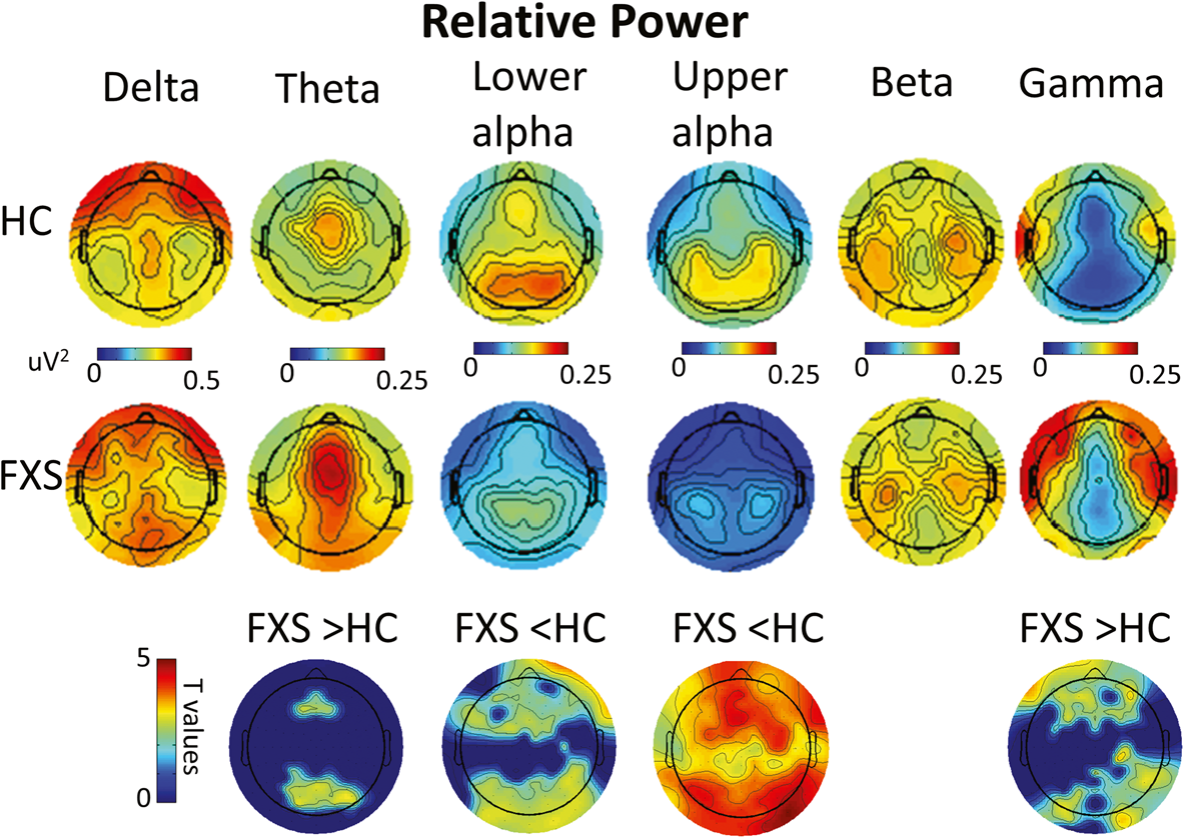
**a** Significant group differences in connectivity strength between FXS and healthy control participants based on permutation tests (*p* < 0.05) show increased connectivity in FXS in the gamma band across electrodes but reduced within-band connectivity in the alpha (*lower* and *upper*) and beta range. **b** Mean and standard error of between-electrode distances for electrode pairs showing group differences (plotted in **a**) in lower alpha, upper alpha, beta and gamma bands. *Asterisk* denotes significant differences in connectivity distances with significant group differences between bands at *p* < 0.05. **c** Bivariate scatter plots depicting the relationship between average connectivity strength (dbWPLI) and average between-electrode distance for FXS (*red dots*) and healthy control participants (*black dots*)

We then evaluated the channel pair distribution for each frequency band showing group differences (Fig. 1) by calculating Euclidean distances between channel pairs based on the 3D position coordinates of electrodes for BioSemi headcaps (Biosemi Instrumentations, Amsterdam, Netherlands), with distances normalized (maximum distance was set to 1). This was done to determine if there was a pattern of altered connectivity between groups specific to short- or long-range connections.

### Cross-frequency amplitude coupling

**Fig. 2:**
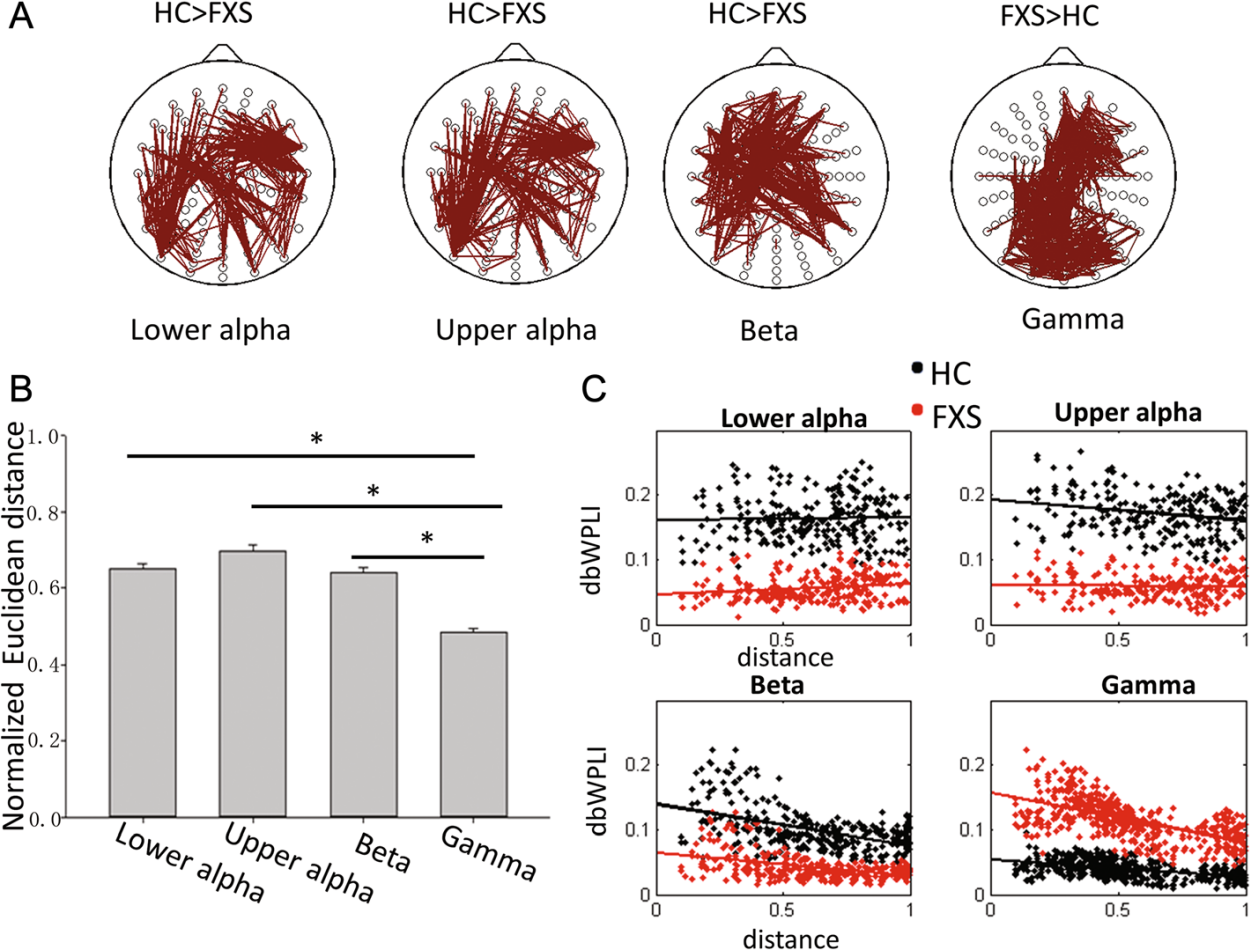
Scalp topographies of relative power spectrum for FXS and healthy control participants per frequency band, with significant group differences presented in the bottom row (*p* < 0.05, corrected). Relative power represents the percentage of power in each frequency band divided by total power across 1–80 Hz

To investigate potential associations of alpha and theta activity with gamma activity, we evaluated cross-frequency amplitude coupling over time. Cross-frequency coupling refers to dependence between electrophysiological activities in different frequency bands [37]. Time series in each electrode were segmented into 2-s epochs. Relative lower and upper alpha, theta, and gamma power were calculated for each epoch. After that, two kinds of correlations were computed between the epoch-by-epoch gamma power with alpha (lower and upper) and theta power [38]. First, correlations were computed in data from every electrode to investigate the local interaction among frequency bands, which we referred as “local coupling” (Additional file 2: Figure S1). Second, correlations were computed between mean power of electrode clusters at the anterior locus showing maximum relative theta power and at the posterior locus showing maximum relative alpha power based on topographies of EEG power (Fig. 2) with gamma power in all other electrodes, which we refer to as “global coupling”. This was done to investigate the association of gamma power in an electrode with activity in alpha and theta bands in other electrodes, the latter both being believed to exert inhibitory modulation over distant brain regions [22]. To correct for multiple comparisons and evaluate differences between control and FXS groups, the correlation coefficients in each electrode and for each subject were transformed to Z scores via Fisher’s Z-transform and then evaluated with a cluster-based permutation test in the Mass Univariate ERP Toolbox for statistical comparisons (5000 permutations [34]).

**Fig. 3:**
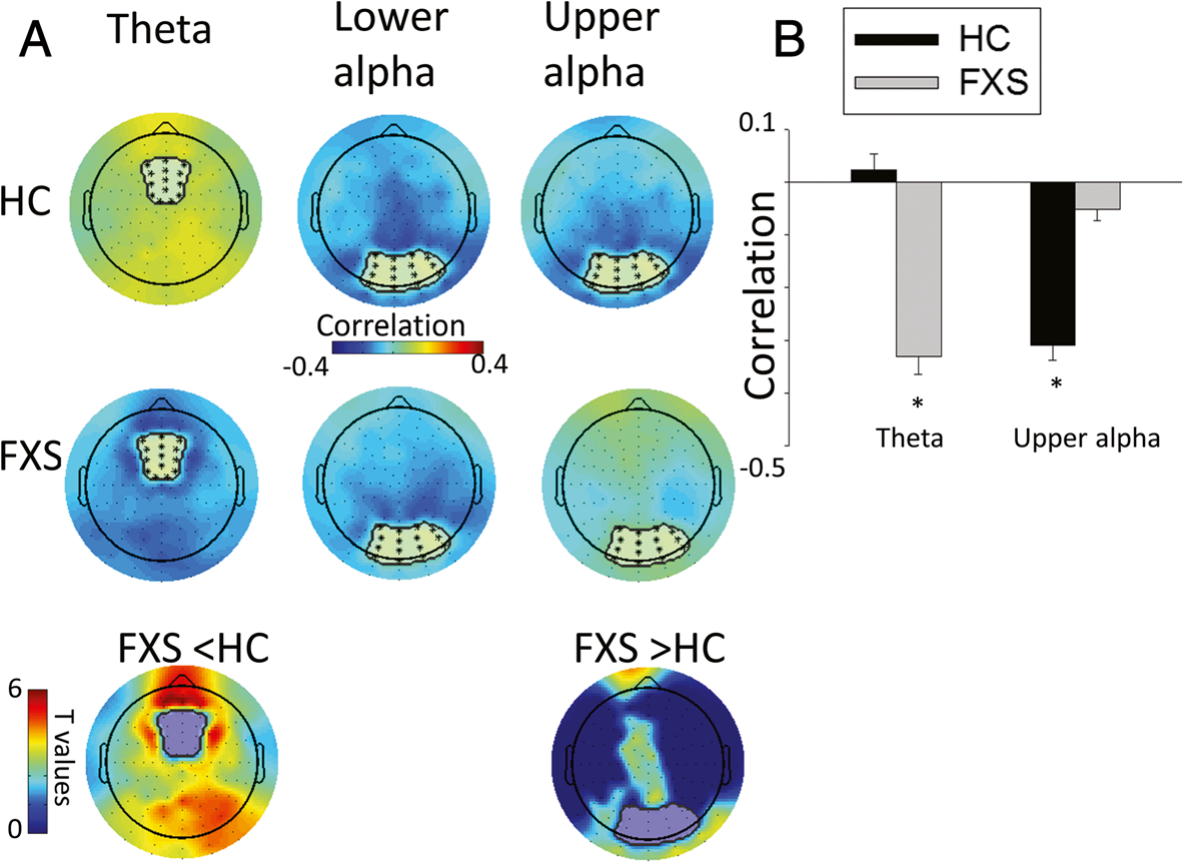
**a** Scalp topographies of “global coupling” showing correlations between activity in the region showing the maximum relative power of activity in the theta, and lower and upper alpha power bands defined as the average of the power in that region of electrodes clusters (marked with *) and gamma power in all other electrodes for FXS and healthy control participants. Significant group differences are presented in the bottom row (*p* < 0.05, corrected), with dark blue reflecting no group difference. **b** Mean and standard error of correlations for all electrodes showing group differences as are plotted in A. *Asterisk* denotes correlations of spectral power in theta and upper alpha bands with gamma band power that are significantly different from zero based on the results of permutation analyses at *p* < 0.05

For correlations showing significant differences between controls and FXS patients (Fig. 3a, Additional file 2: Figure S1A), we also tested whether they were significantly different from zero using a non-parametric permutation approach. To obtain a null distribution for these exploratory correlational analyses, epoch order of gamma power was shuffled while keeping the same epoch order for alpha and theta power. Correlations between alpha and gamma and between theta and gamma were computed for each permutation and repeated 2000 times providing a distribution of correlation values. The correlation for each subject was considered statistically significant if it was beyond the 95th percentile of this distribution.

## Results

### EEG power

Cluster-permutation testing showed stronger relative activity in FXS compared to controls in the theta and gamma frequency bands, with clusters of significant group differences seen in the frontal and occipital regions (Fig. 2). In addition, two way anova (group X frequency [30-80Hz]) showed no interaction between group and frequency. So the magnitude of group differences did not vary across the gamma band (30-80Hz). FXS patients also showed reduced lower alpha band activity than controls in frontal and occipital regions, while reduced upper alpha activity was widely distributed (cluster covering most electrodes). There were no differences between FXS and control participants in delta or beta frequency band power.

### Functional connectivity

The pattern of resting state functional connectivity results is illustrated in Fig. 1. The FXS group showed reduced connectivity in lower alpha, upper alpha, and beta frequency bands, but increased connectivity in the gamma frequency band (Fig. 1a). There were no group differences in within frequency band connectivity for delta or theta band activity.

Paired *t* tests (Fig. 1b) showed that distances between electrode pairs with significant group differences in phase synchronized connectivity were significantly shorter in the gamma band where connectivity was increased in FXS than in lower alpha, upper alpha, and beta bands where connectivity in FXS was reduced. In each frequency band, a linear regression was computed with degree of connectivity as the dependent variable and with between-electrode distance, group (FXS, Controls), and their interaction as independent variables (Fig. 1c). Results indicated no significant effects in the lower alpha band. In the upper alpha band, there was a significant interaction between group and electrode distance (*t* = 3.0, *p* = 0.003). Upper alpha connectivity in controls decreased with increased electrode distance (*t* = 3.6, *p* < 0.001); this effect was not seen in FXS. In the beta band, both between-electrode distance (*t* = 12.0, *p* < 0.001) and its interaction with group (*t* = 3.8, *p* < 0.001) were significant. Although both groups showed decreased beta connectivity with increased between-electrode distance, the effect was significantly reduced in FXS patients. In the gamma band, both between-electrode distance (*t* = 7.9, *p* < 0.001) and its interaction with group (*t* = 9.9, *p* < 0.001) were highly significant. Although both groups showed decreased gamma connectivity with greater between-electrode distance, the FXS group showed stronger effects. Because connectivity at a scale on the order of even short electrode distances is well beyond the scale maintained by individual PV positive inhibitory neurons, the observation of increased functional connectivity of gamma band activity in FXS across a variety of inter-electrode distances indicates a greater spread of coherent high frequency neural activity in FXS than in controls.

### Cross-frequency amplitude coupling

When evaluating amplitude coupling across electrodes, cluster-permutation testing showed that the inverse correlation between upper alpha and gamma power was significantly reduced in FXS patients compared to controls in the occipital, parietal, and frontal regions (Fig. 3a). There were no lower alpha to gamma power correlation differences between FXS and controls. The opposite pattern was seen in the theta band, where negative theta to gamma power correlations were stronger in FXS compared to controls, with clusters of significant group differences in cross-frequency coupling seen in the occipital, parietal, and frontal regions. Follow-up permutation tests showed that negative theta to gamma power correlation was significantly greater than zero only in FXS participants, while the negative upper alpha to gamma power correlation was significant only in controls (Fig. 3b). For amplitude coupling within individual electrodes, performed to examine local circuitry effects, cluster-permutation testing showed a similar pattern with stronger negative theta to gamma power correlation, but reduced upper alpha to gamma power correlation in FXS compared to controls (Additional file 2: Figure S1 and Additional file 3: Figure S2).

These results suggest that while upper alpha power negatively coupled with gamma power both within and across electrodes in controls, FXS patients displayed excessive gamma activity and reduced upper alpha power without showing a coupling of gamma and upper alpha band activity. In other words, reduced upper alpha-related inhibition of gamma power was observed in FXS. Instead, FXS showed stronger negative theta to gamma power coupling both within and across electrodes. Follow-up phase amplitude coupling analyses were not significant (see Additional file 4).

### Severity of gamma abnormalities in FXS patients

**Fig. 4:**
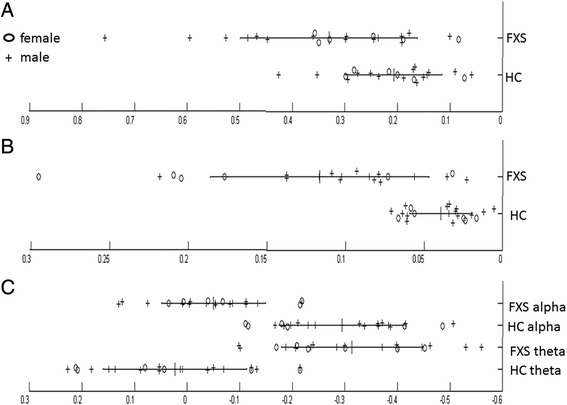
Scatter plot of each participant’s values on three gamma measurements for FXS and healthy control participants: **a** gamma power, **b** gamma connectivity, and **c** amplitude coupling of gamma with theta and alpha band activity across electrodes. *Circle* denotes female participants, and *plus sign* denotes male participants

In order to characterize the prevalence of the different abnormalities we observed, we computed individual participant values for each parameter showing significant abnormalities in FXS patients, including alterations in gamma activity in power, functional connectivity, and cross-frequency amplitude coupling (Fig. 4). FXS participants showed variable levels of increased gamma power, with somewhat greater group separation in gamma connectivity indices. Amplitude coupling measurements more robustly and consistently separated FXS and healthy study participants. For example, while only approximately 50% of FXS patients had gamma power levels increased more than 1 SD beyond healthy controls, almost every FXS participant had altered long distance coupling (outside the range of normal values) of local gamma with local and global upper alpha and theta activity. The heterogeneity in gamma power is noteworthy since all FXS participants had documented full mutations. The findings of altered long distance functional connectivity suggest that different patterns of cortico-cortical connectivity may be an important factor contributing to neural hyperexcitability reflected in increased gamma power in FXS.

### Correlations with demographic and clinical variables

Exploratory correlational analyses were performed between EEG measurements showing significant alteration in FXS and SCQ and Adolescent/Adult Sensory Profile scores in FXS participants. Increased resting gamma power was significantly correlated with social-communication abnormalities as assessed with the SCQ (*r* = 0.56, *p* = 0.03). Lower alpha power was significantly correlated with greater social impairment in SCQ scores (*r* = −0.58, *p* = 0.02) and with hypersensitivity to sensory stimuli on the Sensory Profile scale (*r* = −0.51, *p* = 0.03). For functional connectivity and cross-frequency coupling measurements, no correlations with clinical variables were significant. Within each group, no significant correlations for EEG parameters were observed with age or gender. The consistent and marked reductions in IQ, especially in male FXS patients (all with IQ between 40 and 50), made correlations with electrophysiological parameters difficult to meaningfully evaluate.

### Sex differences in FXS

The FXS full mutation in males is typically associated with a more severe profile of intellectual and behavioral deficits due to females having a compensatory functional FMR1 gene on their typically unaffected X chromosome. Therefore, it is important to consider gender effects in our analyses. We repeated all of the above analyses in two ways. First, we analyzed the male only group and all reported effects remained significant (see Additional file 5: Figure S3, Additional file 6: Figure S4, Additional file 7: Figure S5, and Additional file 8: Figure S6). Second, we compared male and female participants and did not find significant sex differences (see Additional file 9: Figure S7, though power to detect such effects was not high).

## Discussion

This case-control study investigated multiple aspects of brain system function in the largest sample of non-epileptic, full mutation FXS patients studied to date with quantitative dense-array resting-state EEG. Abnormalities were evident across measures of spectral power, functional connectivity, and cross-frequency amplitude coupling and were consistent with predictions based on the *fmr1* KO mouse electrophysiology [7, 20, 24]. First, alterations in gamma band activity involved increased relative gamma power and an increased coherence of gamma band activity across nearby electrodes. This pattern is consistent with an imbalance of excitatory over inhibitory activity in FXS. The associations of electrophysiological alterations with abnormalities in social function and sensory sensitivities provide the first evidence of the clinical relevance of quantitative EEG findings in this population. Secondly, relative to controls, individuals with FXS showed reduced gamma amplitude coupling with upper alpha band activity but increased coupling with theta activity. As alpha and theta band activity are believed to exert top down inhibitory and regulatory modulation of sensory systems, these observations provide novel evidence that hyperexcitability in sensory cortex involves not only altered local circuit dysfunction in the interaction of interneurons and pyramidal cells as demonstrated in the fmr1 KO mouse [7], but also alterations in the long distance functional connectivity from association cortex and thalamus to sensory cortex known to regulate local circuit excitability. While there was better group separation on the amplitude coupling indices, clinical correlations were significant only with the power of resting gamma alterations. This pattern of findings suggests that variable disease-related disturbances and compensatory adaptations may leave FXS patients with a net level of residual neural hyperexcitability reflected in elevated gamma power that determines important aspects of their level of neurological and functional disability. These findings provide new mechanistic understanding of cortical hyperexcitability in FXS, involving increased high-frequency local circuit activity that varied in relation to what appear to be abnormal and compensatory long-distance functional connectivity. They also suggest a potential utility of resting state EEG alterations as translational biomarkers for clinically relevant aspects of FXS biology for identifying individual FXS patients likely to benefit from treatments aimed at reducing neocortical hyperexcitability and for tracking those effects.

The U-shaped pattern of relative EEG power in individuals with FXS (Fig. 2) is similar in form to one we described previously in autism [39]. Compared to healthy age-matched control participants, FXS patients showed enhanced power in lower (theta) and higher (gamma) bands, but reduced power in intermediate low and high alpha bands. Elevated theta and reduced alpha power have been reported previously in FXS [15], but the enhanced power in the gamma band and its clinical relevance have not been previously described. This enhanced gamma power is consistent with studies of *fmr1* knockout mice demonstrating heightened neuronal excitability related to alterations in input to fast-spiking inhibitory interneurons that synchronize and control high-frequency gamma band neural activity [4, 40]. A recent study in wild-type rats showed that enhanced gamma oscillation was observed when NMDA-receptor blockade was established [41]. NMDA-receptor hypofunction has been reported in several studies with *fmr1* knockout mice [42–44] and thus may be one contributing factor for the gamma band alterations observed in the present study.

Our functional connectivity analyses revealed reduced long-range functional connectivity in the alpha and beta bands, but enhanced shorter-range connectivity in the gamma band in FXS. The decreased functional connectivity in the alpha and beta bands parallels previous findings in FXS [16]. We did not however observe the increased connectivity within the theta band that has been reported, though we did observe increased theta gamma coupling. For the first time, we report increased connectivity in gamma band activity in FXS patients. A previous fmr1 KO mouse study examined cross-frequency theta-gamma coupling, but during a cognitive task and when computed in the same hippocampal electrode [24]. While related to focus of the present study, the differences in species, in rest vs task performance situations, and within rather than across distant brain sites are differences that future work will need to resolve to integrate the observations. Further translational work bridging preclinical and clinical findings is needed to more directly link clinical neurophysiological findings to observations seen in fmr1 KO mice.

Our observation of increased spatial extent of coherent gamma band activity across more distant electrode pairs suggests an increased cortical spread of neural excitability paralleling effects observed in slice preparation data but on a far greater spatial scale [7]. It aligns with observations of increased neural synchrony observed in *fmr1* knockout mice during sleep and quiet wakefulness [6]. The pattern of reduced long range connectivity in the alpha and beta bands suggests that reduced top-down inhibitory regulation of neocortical sensory systems may contribute to increased neural excitability of sensory cortex in FXS.

Given the alterations we observed in EEG power and functional connectivity in FXS, we investigated the coupling between low frequency band activity that was abnormal (alpha and theta activity) and high-frequency gamma band activity both within and across electrodes. We did this to determine whether the pattern of findings seen in gamma power and connectivity was related to a disruption in top-down modulation. Based on EEG data, it is not possible to determine whether a local circuit dysfunction is causing or resulting from the altered pattern of reduced long-distance functional connectivity in FXS. However, previous studies have shown that low frequency oscillations (e.g., alpha, theta) provide top-down inhibitory and modulatory influences in large, distributed neural networks, whereas fast oscillations (e.g., gamma) at rest are more related to neurophysiological tone in local networks [45]. Thus, the reduced alpha power, connectivity, and amplitude coupling in FXS may represent a failure of top-down modulation provided by alpha band input that could reduce gamma power in sensory systems. Previous studies have characterized the functional role of alpha band activity as actively inhibiting the processing of sensory information at rest and when environmental cues are not task relevant [22]. Posterior alpha has been reported to provide top-down control especially in visual attention studies, and thalamus has been identified as an important source of cortical alpha oscillations [46]. Both simulation and experimental studies have demonstrated that lower frequency oscillation rhythms (e.g., alpha, theta) can sustain long-range synchronization [45, 47], while synchronous activity in higher oscillation rhythms (e.g., gamma) declines more rapidly with increasing distances [48]. As a result, slower oscillations are better suited for top-down modulation by synchronizing and organizing activity across different brain regions [45]. This is consistent with findings from a nonhuman primate study of V1 and V4 showing that gamma rhythms propagate in a feedforward fashion from early to higher level visual processing regions, whereas alpha rhythms propagate in a feedback fashion to primary visual cortex [49].

In contrast to the reduced upper alpha-to-gamma coupling, FXS showed strong theta-to-gamma coupling with atypical theta connectivity being related to lower levels of gamma power (Fig. 3). Thus, while alpha power and coupling were reduced, theta power and coupling were increased in FXS, indicating a fundamental alteration in the pattern of cortico-cortical connectivity that supports top-down modulation in sensory systems. Further clinical and preclinical studies are needed to fully clarify the meaning of this novel observation, but one possibility is that in the context of reduced inhibitory modulation of alpha oscillations on gamma band activity, a second long-distance regulatory circuitry operating in the theta band may be relied upon to downregulate high-frequency neural activity in the gamma band, a compensation which is only partially and variably successful given the observation of clinically relevant increased gamma power in FXS. As theta power phasically synchronizes neural activity across brain regions to support different types of higher level cognition [50], a tonic activation to suppress sensory hyperexcitability reflected in increased theta power at rest might limit that phasic modulation and thereby contribute to the severe intellectual limitations often seen in FXS. Reduced alpha power might also contribute to the severe intellectual limitation since alpha activity has been reported to positively correlate with cognitive parameters [51].

The functional role of theta oscillation is related to its neural sources in the prefrontal and anterior cingulate cortex (ACC) [52], which play important roles in inhibitory control of behavior, behavioral flexibility, and error monitoring [53, 54]. During tasks requiring top-down inhibition of attention or behavior, microelectrode recordings in superficial cingulate layers exhibit strong task-related theta activity [55]. In addition, intermittent theta-burst stimulation has been shown to increase cortical inhibition in rat neocortex by reducing parvalbumin expression in fast-spiking interneurons [56], and stimulation in theta frequency bands increases expression of GABA precursors in inhibitory cortical systems [57]. Our gamma amplitude coupling associations may represent neural system factors that in vivo impact local circuit neurophysiology known to be altered in FXS, such as alterations in metabotropic glutamate receptor (mGluR) activation believed to be a cause of neuronal hyperexcitability in FXS. Inhibitory interneurons in mouse neocortex have been reported to fire in the theta frequency during mGluR activation [44, 58]. In addition, *FMRP* is highly expressed in the hippocampus [59], and long-term potentiation (LTP) elicited by theta burst stimulation has been reported to be impaired in the CA1 hippocampal subfield in *fmr1* KO mice [60].

Interest in systems biology alterations to complement understanding of local circuit pathology may be important not only for comprehensive models of pathology in FXS, but because individual variability in system-level modulatory factors may contribute to the wide range of clinical phenotypes seen even in patients with full mutation. This variability might explain the inconsistent treatment response to drugs targeting mGluR and other mechanisms aiming to reduce neuronal hyperexcitability, which have had more consistently positive effects in animal models. In this context, our findings not only provide new mechanistic understanding of FXS but also suggest that EEG studies of FXS may provide biomarkers for delineating disease heterogeneity and predicting and tracking response in human and mouse models to drugs targeting neuronal hyperactivity. Such approaches are urgently needed to advance drug development and personalized medicine for FXS patients. Our observation that quantitative EEG alterations were related to the severity of social communication and sensory reactivity problems supports the potential clinical utility of this approach.

This study has certain limitations, including the wide age range (12–57 years old) and the fact that younger children were not assessed. Although FXS and control groups were age-matched and no significant age effects were observed in the data, studies with younger populations remain an important target for future research. Other effects such as sex differences and medication effects were not statistically significant, but further research is needed to address those issues. Third, our study did not compare resting state abnormalities in FXS directly with other developmental disabilities to establish specificity of deficits to FXS relative to general effects of intellectual or developmental disability. While the parallel findings from our study and preclinical work in *fmr1* KO mice suggest a relevance to FXS, more research is needed to determine whether similar findings might be seen in the subset of ASD patients who demonstrate sensory hypersensitivities or other signs of cortical excitability, as well as in multiple other neurodevelopmental disorders.

## Conclusions

In summary, we found an abnormal U-shaped alteration of spectral power with reduced long-range inhibitory and enhanced excitatory shorter-range connectivity in FXS. Furthermore, we found stronger theta-to-gamma amplitude coupling in FXS, possibly serving as a compensatory response to reduced top-down alpha-band inhibitory modulation and intrinsic pathology of local neural circuit excitability in sensory systems. Taken in combination, our findings provide direct in vivo evidence in FXS patients of heightened cortical arousal, reduced top-down regulatory input in the alpha band, and increased modulation in the theta band that together may determine the level of circuit hyper-excitability in sensory cortex in vivo.

## Additional files

Additional file 1: Table S1. EEG measurements in the combined FXS group and in both the medicated FXS and non-medicated FXS group. (DOCX 14 kb)
Additional file 2: Figure S1. (A) Scalp topographies of “local coupling”, showing correlations in each electrode between relative power of activity in the theta, and lower and upper alpha power bands and gamma power for FXS and healthy control participants, with significant group differences presented in the bottom row (*p* < 0.05, corrected), with dark blue reflecting no group difference. (B) Mean and standard error of correlations for all electrodes showing group differences as are plotted in A. * denotes correlations of spectral power in theta and upper alpha bands with gamma band power that are significantly different from zero based on the results of permutation analyses at *p* < 0.05. (TIF 3754 kb)
Additional file 3: Figure S2. Scatter plot of each participant’s values of coupling with gamma in theta and alpha band within electrodes for FXS and healthy control participants. Circle denotes female participants, plus (+) denotes male participants. (TIF 321 kb)
Additional file 4: Cross-frequency amplitude coupling within individual electrodes. (DOCX 19 kb)
Additional file 5: Figure S3. Scalp topographies of relative power spectrum are presented for *male* FXS and *male* healthy control participants per frequency band, with significant group differences presented in the bottom row (*p* < 0.05, corrected). Relative power represents the percentage of power in each frequency band divided by total power across 1–80 Hz. (TIF 5063 kb)
Additional file 6: Figure S4. (A) Significant group differences in connectivity strength between *male* FXS and *male* healthy control participants based on permutation tests (*p* < 0.05) show increased connectivity in FXS in the gamma band but reduced connectivity in the alpha (lower and upper) and beta range. (B) Mean and standard error of between-electrode distances for electrode pairs showing group differences (plotted in A) in lower alpha, upper alpha, beta, and gamma bands. * denotes significant differences in connectivity distances with significant group differences between bands at *p* < 0.05. (C) Bivariate scatter plots depict the relationship between averaged connectivity strength (dbWPLI) and averaged between-electrode distance for FXS (red dots) and Healthy Control participants (black dots). (TIF 3314 kb)
Additional file 7: Figure S5. (A) Scalp topographies of “global coupling”, showing correlations between activity in the region showing the maximum relative power of activity in the theta, and lower and upper alpha power bands defined as the average of the power in that region of electrodes clusters (marked with *) and gamma power in all other electrodes for *male* FXS and *male* healthy control participants. Significant group differences are presented in the bottom row (*p* < 0.05, corrected), with dark blue reflecting no group difference. (B) Mean and standard error of correlations for all electrodes showing group differences as are plotted in A. * denotes correlations of spectral power in theta and upper alpha bands with gamma band power that are significantly different from zero based on the results of permutation analyses at *p* < 0.05. (TIF 3306 kb)
Additional file 8: Figure S6 (A) Scalp topographies of “local coupling”, showing correlations in each electrode between relative power of activity in the theta, and lower and upper alpha power bands and gamma power for *male* FXS and *male* healthy control participants, with significant group differences presented in the bottom row (*p* < 0.05, corrected), with dark blue reflecting no group difference. (B) Mean and standard error of correlations for all electrodes showing group differences as are plotted in A. * denotes correlations of spectral power in theta and upper alpha bands with gamma band power that are significantly different from zero based on the results of permutation analyses at *p* < 0.05. (TIF 4297 kb)
Additional file 9: Figure S7. Scatter plots of each participant’s values of power and connectivity in delta, theta, lower alpha, upper alpha, beta bands for FXS and healthy control participants. Circle denotes female participants, plus denotes male participants. (TIF 475 kb)
