# Supplementary material for: A resting EEG study of neocortical hyperexcitability and altered functional connectivity in fragile X syndrome
Source: J Neurodev Disord. 2017 Mar 14;9:11. doi: 10.1186/s11689-017-9191-z (PMC5351111; doi:10.1186/s11689-017-9191-z)
Supplement: Additional file 1: Table S1. — EEG measurements in the combined FXS group and in both the medicated FXS and non-medicated FXS group. (DOCX 14 kb) [file 11689_2017_9191_MOESM1_ESM.docx]

Supplemental table 1

EEG measurements in the combined FXS group and in both the medicated FXS and non-medicated FXS group

|  | Combined group (N=21)  Mean(SD) | Medicated group (N=9)  Mean(SD) | Non-medicated group (N=12)  Mean(SD) | Medicated vs. Non-medicated |
| --- | --- | --- | --- | --- |
| delta power | 0.35(0.10) | 0.33(0.08) | 0.36(0.12) | t=-0.55 p=0.59 |
| theta power | 0.25(0.12) | 0.22(0.04) | 0.27(0.16) | t=-0.93 p=0.36 |
| lower alpha power | 0.08(0.05) | 0.06(0.03) | 0.09(0.06) | t=-1.64 p=0.11 |
| upper alpha power | 0.06(0.03) | 0.04(0.02) | 0.06(0.04) | t=-1.62 p=0.12 |
| beta power | 0.15(0.06) | 0.17(0.05) | 0.14(0.07) | t=0.90 p=0.38 |
| gamma power | 0.33(0.17) | 0.37(0.22) | 0.30(0.12) | t=1.0 p=0.33 |
| delta connectivity | 0.05(0.04) | 0.04(0.02) | 0.05(0.04) | t=-0.72 p=0.48 |
| theta connectivity | 0.06(0.05) | 0.05(0.03) | 0.08(0.06) | t=-1.50 p=0.15 |
| lower alpha connectivity | 0.06(0.03) | 0.05(0.03) | 0.06(0.03) | t=-0.25 p=0.80 |
| upper alpha connectivity | 0.06(0.04) | 0.06(0.03) | 0.06(0.04) | t=-0.1 p=0.92 |
| beta connectivity | 0.04(0.02) | 0.04(0.01) | 0.04(0.02) | t=0.35 p=0.73 |
| gamma connectivity | 0.12(0.07) | 0.13(0.06) | 0.11(0.08) | t=0.64 p=0.53 |
| theta-gamma coupling across electrodes | -0.31(0.13) | -0.31(0.15) | -0.31(0.13) | t=0.02 p=0.98 |
| upper alpha-gamma coupling across electrodes | -0.05(0.1) | -0.08(0.07) | -0.02(0.11) | t=-1.41 p=0.17 |
| theta-gamma coupling within electrodes | -0.38(0.14) | -0.41(0.16) | -0.36(0.14) | t=-0.96 p=0.35 |
| upper alpha-gamma coupling within electrodes | -0.01(0.11) | -0.02(0.06) | 0.03(0.13) | t=-1.14 p=0.27 |
