# Supplementary figures and images for: A resting EEG study of neocortical hyperexcitability and altered functional connectivity in fragile X syndrome
Source: J Neurodev Disord. 2017 Mar 14;9:11. doi: 10.1186/s11689-017-9191-z (PMC5351111; doi:10.1186/s11689-017-9191-z)

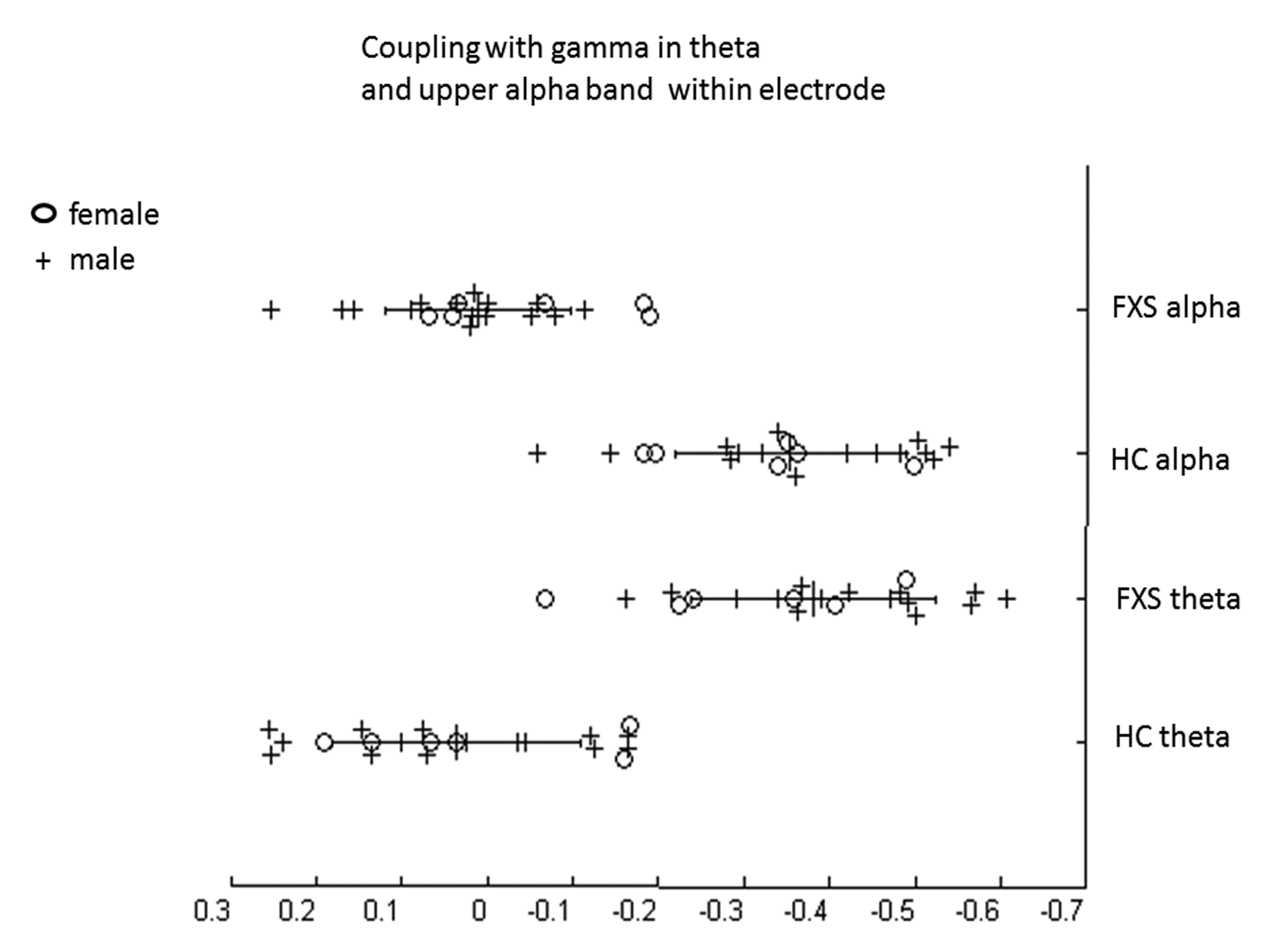

Supplement: Additional file 3: Figure S2. — Scatter plot of each participant’s values of coupling with gamma in theta and alpha band within electrodes for FXS and healthy control participants. Circle denotes female participants, plus (+) denotes male participants. (TIF 321 kb) [file 11689_2017_9191_MOESM3_ESM.tif]
