# Supplementary material for: A resting EEG study of neocortical hyperexcitability and altered functional connectivity in fragile X syndrome
Source: J Neurodev Disord. 2017 Mar 14;9:11. doi: 10.1186/s11689-017-9191-z (PMC5351111; doi:10.1186/s11689-017-9191-z)
Supplement: Additional file 4: — Cross-frequency amplitude coupling within individual electrodes. (DOCX 19 kb) [file 11689_2017_9191_MOESM4_ESM.docx]

**Cross-frequency amplitude coupling within individual electrodes**

For amplitude coupling within individual electrodes, cluster-permutation testing (Supplemental Figure 1A) showed a reduced negative upper alpha to gamma power correlation in FXS, with clusters of significant group differences seen in the occipital region. The opposite pattern was seen in the theta band, where stronger negative theta to gamma power correlation in FXS compared to controls was seen in in frontal and occipital regions. Permutation tests (Supplemental Figure 1B) showed that the only statistically significant different from zero associations were the negative theta to gamma power correlation in FXS and negative upper alpha to gamma power correlation in controls.

**Additional scatterplots for all other measurements**

Supplemental Figure 3 shows scatterplots for measurements not presented in this way in the paper, including power and connectivity in delta, theta, lower alpha, upper alpha, beta bands.

**Phase-amplitude coupling**

To investigate phase-amplitude coupling between phase of low frequency (theta, lower alpha, and upper alpha) and amplitude of high frequency (gamma), entire EEG time series in each electrode and for each participant was filtered separately in theta (3-7Hz), lower alpha (8-10Hz), upper alpha (10-12Hz) and gamma bands (30-80Hz). Then the phase of slow frequency (theta, lower alpha and upper alpha) activity and the amplitude of fast frequency (gamma) activity were extracted and represented as vectors, in which phase of slow frequency activity was represented as a vector angle while amplitude of fast frequency activity was represented as vector length, with mean vector length computed as a Modulation Index (Canolty 2006). The Modulation Index (MI) measure was implemented in a PAC plugin in EEGLAB toolbox (Delorme and Makeig 2004). As a result, for each participant and in each electrode, we obtained MI for theta-gamma PAC, lower alpha-gamma PAC and upper alpha-gamma PAC. To correct for multiple comparisons and evaluate differences between control and FXS groups, MI in each electrode and for each subject was evaluated with a cluster-based permutation test in the Mass Univariate ERP Toolbox for statistical comparisons (5000 permutations, Groppe et al., 2011). The results did not show significant group differences in either theta-gamma PAC, lower alpha-gamma PAC or upper alpha-gamma PAC. Therefore, we did not identify alterations in our resting state study in PAC, unlike previous fmr1 KO mouse model work showing altered theta-gamma PAC in hippocampus during a cognitive task (Radwan et al., 2016).

**Results with Male only participants**

Supplemental Figures 4-7 show power, functional connectivity and cross frequency coupling results for the 15 male FXS and 15 male control participants (analyses conducted only with the male participants). The figures showed similar pattern as the analyses of all participants as reported in the paper as indicated in those figures.
